# A Silent Left Atrial Myxoma: A Rare Benign Cardiac Tumor

**DOI:** 10.7759/cureus.2551

**Published:** 2018-04-30

**Authors:** Rizwan Ali, Arooj Tahir, Muhammad Nadeem, Syed B Rizvi

**Affiliations:** 1 Internal Medicine, Rapides Regional Hospital, Alexandria, USA; 2 Medicine, St Francis Medical Center; 3 Cardiology Department, Rapides Regional Hospital, Alexandria, USA

**Keywords:** atrial myxoma, cardiac tumor

## Abstract

Primary cardiac tumors are extremely rare. In one study, incidence was reported being less than 0.1%. The purpose of this case report is to review different presentations of cardiac myxoma. A 34-year-old female with past medical history of drug abuse was brought into the emergency department (ED) after a motor vehicle collision. She was found to have multiple fractures with a hypodense mass in the left atrium. Further evaluation showed a left atrial myxoma. The patient underwent myxoma resection. The clinical appearance of myxoma varies from non-specific to life-threatening complications, such as stroke, acute heart failure, or even sudden death.

A surgical resection is the treatment of choice for cardiac myxomas.

## Introduction

Primary cardiac tumors are extremely rare [1]. In one study, incidence was reported less than 0.1% [2]. However, metastatic involvement of the heart is over 20 times more common and has been reported in an autopsy series in up to one in five patients dying of cancer [3]. Most heart tumors are benign. Nearly half of the benign heart tumors are myxomas [3]. Clinical appearance varies from non-specific to life-threatening complications, such as stroke, acute heart failure, or even sudden death [4-7]. Diagnosis and subsequent surgical treatment strongly depend on the clinical symptoms, but their extent does not correlate with the risk for serious complications [4].

## Case presentation

**Figure 1:**
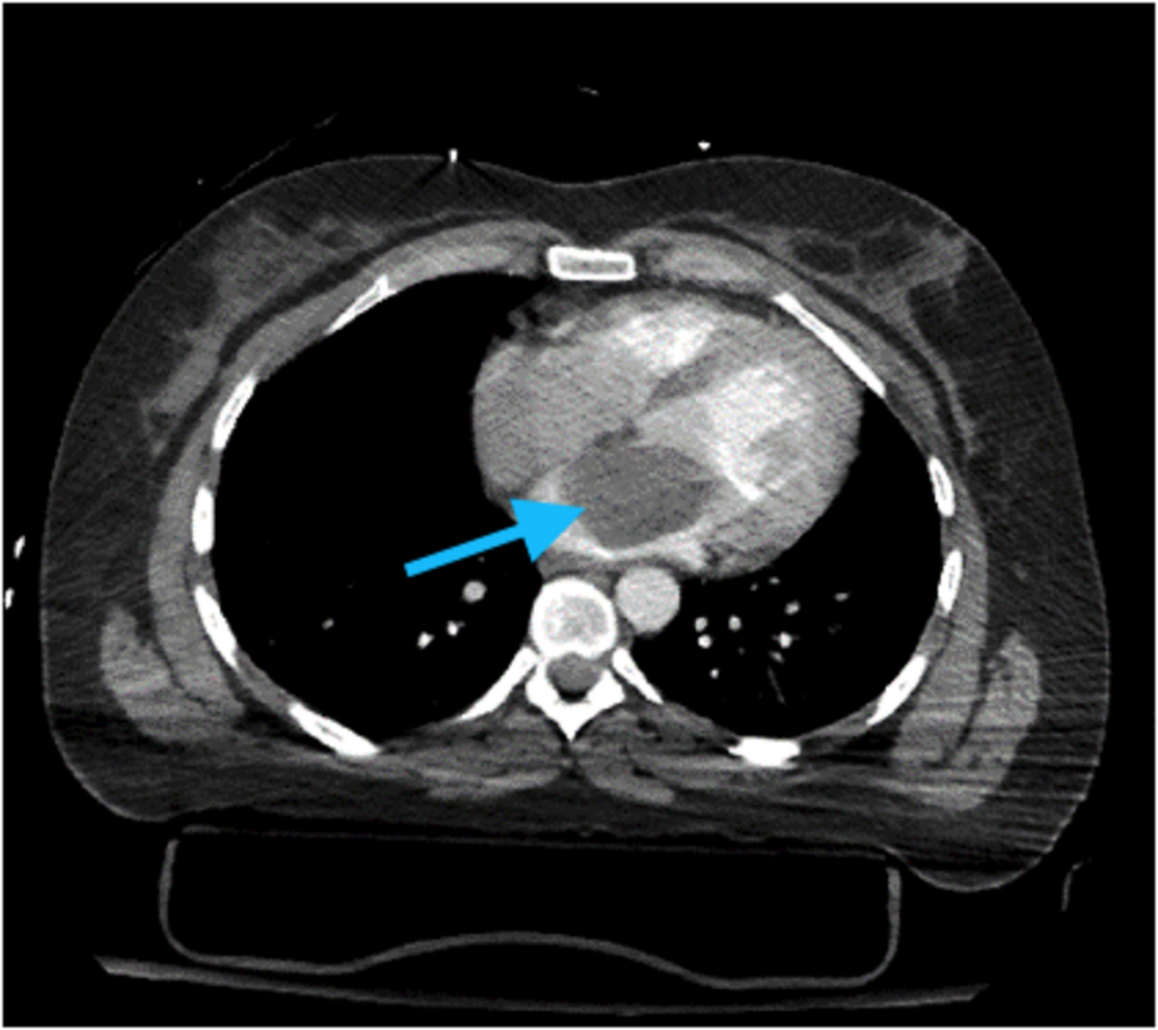
Computed tomography (CT) scan of Chest Blue arrow pointing towards hypodense mass in left atrium

A 34-year-old female with a past medical history of drug abuse was brought into the emergency department (ED) after a motor vehicle collision; the patient was driving a jeep. The patient was confused in the ED. Initial imaging showed a closed distal right tibial fracture, open distal right fibular fracture, and a Grade 3 open right talus and calcaneus fracture. A computed tomography (CT) scan of the chest showed large hypodense mass within the left atrium of the heart (Figure 1).

The patient was emergently taken to the operation theater for an open fracture repair. Initially, intramedullary nailing of the right tibial shaft fracture and incision and debridement of the open right fibular shaft, calcaneus, and talus fractures were done. Multiplanar right ankle spanning external fixation was applied. Wound vacuum-assisted closure (VAC) was also applied to the open wound. The patient subsequently underwent multiple debridements and was placed on broad-spectrum antibiotics but her wound got worse. After a detailed discussion with patient and family, it was decided to proceed with below-the-knee amputation. The patient did develop a wound infection after amputation and continued on broad-spectrum antibiotics.

**Figure 2:**
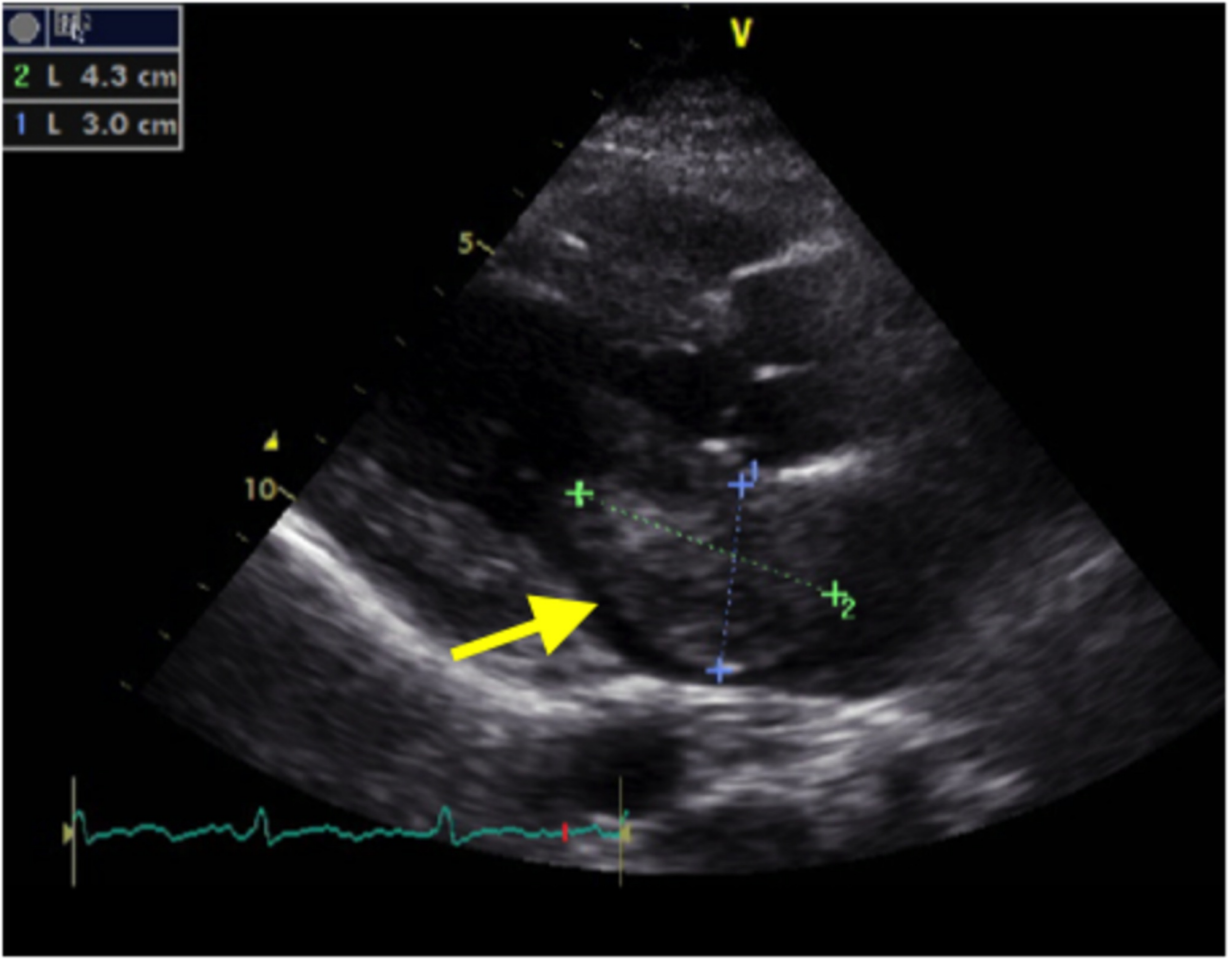
Transthoracic echocardiogram Yellow arrow pointing towards left atrial mass

**Figure 3:**
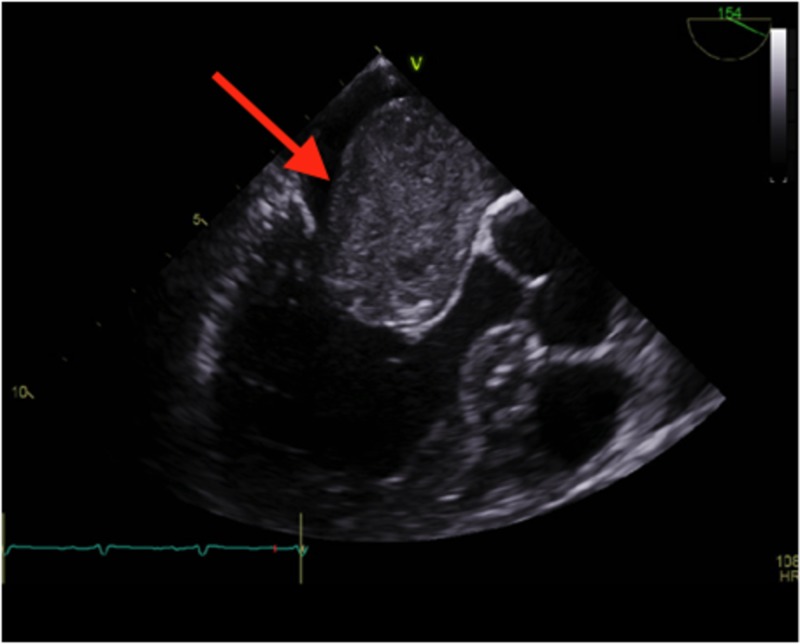
Transesophageal echocardiogram Red arrow showing left atrial mass

For the left atrial mass, cardiology was consulted. The patient underwent transthoracic and transesophageal echocardiograms that confirmed the left atrial mass was consistent with a myxoma (Figures 2-3).

Cardiovascular surgery was consulted for the resection of the left atrial myxoma. The patient underwent an uncomplicated resection of the left atrial mass. Pathology findings were consistent with a myxoma. The patient was discharged with intravenous antibiotics for the left knee wound.

## Discussion

Over 75% of primary cardiac tumors are benign [8-9]. Myxomas are the most common primary cardiac neoplasm. Histologically, these tumors are composed of scattered cells within a mucopolysaccharide stroma. Macroscopically, typical myxomas are pedunculated and gelatinous in consistency; the surface may be smooth, villous, or friable. Tumors vary widely in size, ranging from 1 to 15 cm in diameter, and weigh between 15 and 180 grams [10]. About 35% of myxomas are friable or villous, and these tend to present with emboli. The cardiovascular manifestations depend upon the anatomic location of the tumor. Approximately 80% of myxomas originate in the left atrium, and most of the remainder are found in the right atrium [11-12]. In addition to their cardiovascular effects, patients with myxomas frequently have constitutional symptoms (eg, weight loss, fever) and laboratory abnormalities that suggest the presence of a connective tissue disease [13]. According to one study, the incidence of cardiovascular symptoms was 67% [14]. These symptoms consisted of mitral valve obstruction, electrocardiographic evidence of left atrial hypertrophy, auscultatory findings were found in 64%, and the classic tumor plop was found in only 15%. Systemic embolization was present in 29%; 20% had neurologic deficits. The incidence of embolization was associated with smaller size (< 4.5 cm) and softer tumors [15]. Constitutional symptoms (e.g., fever, weight loss) were seen in 34% of patients. Echocardiography is the simplest technique for such evaluation; cardiac magnetic resonance imaging (MRI) and ultrafast CT provide more detailed information. Tumors that occur from or invade the epicardial surface of the heart require coronary angiography preoperatively to define distortion of the coronary arteries and determine coronary blood supply to the tumor. Once a presumptive diagnosis of myxoma has been made on imaging studies, prompt resection is required because of the risk of embolization or cardiovascular complications, including sudden death [12, 16-17].

## Conclusions

Over 75% of primary cardiac tumors are benign. Myxomas are the most common primary cardiac neoplasm. Myxomas can present with a wide range of symptoms, such as constitutional symptoms, cardiovascular symptoms (including obstruction and heart failure), and neurologic symptoms, such as embolization with infarction. Once one suspects myxomas, imaging studies (echocardiogram, transesophageal echocardiogram, chest CT, and MRI) can be done. Surgical resection is the treatment of the choice.
